# From nasal cytology to cellular endotyping: mast cell–eosinophil interactions and overlapping inflammation in allergic rhinitis

**DOI:** 10.3389/fimmu.2026.1969392

**Published:** 2026-09-23

**Authors:** Matteo Gelardi

**Affiliations:** Unit of Otolaryngology, Department of Clinical and Experimental Medicine, University of Foggia, Foggia, Italy

**Keywords:** allergic rhinitis, cellular endotyping, eosinophils, mast cells, mucosal immunity, nasal cytology, overlapping rhinitis, precision medicine

## Abstract

Allergic rhinitis is traditionally defined by allergen sensitization and type 2 inflammation, yet increasing evidence indicates that this framework does not fully capture the biological heterogeneity of the nasal mucosa. The local inflammatory landscape reflects a dynamic interaction among epithelial cells, innate and adaptive lymphoid populations, mast cells, eosinophils, stromal elements, and vascular components, and may diverge substantially from systemic biomarkers. Recent single-cell and multi-omic studies have further revealed disease-associated alterations in epithelial differentiation, stromal activation, and cell-cell communication, reinforcing the concept of the nasal mucosa as an integrated immunological ecosystem. Within this context, mast cell-eosinophil interactions appear particularly relevant, as their coexistence and degranulation may characterize inflammatory states that differ from isolated eosinophilia. Nasal cytology provides a clinically accessible morphological readout of this local cellular architecture and has shown that allergic rhinitis does not correspond to a single cytological expression. In some patients, established allergic sensitization coexists with recognized non-allergic cellular rhinitis entities, including non-allergic rhinitis with eosinophils (NARES), non-allergic rhinitis with mast cells (NARMA), and non-allergic rhinitis with eosinophils and mast cells (NARESMA), giving rise to the concept of overlapping rhinitis. This phenomenon challenges a binary allergic versus non-allergic classification and may contribute to difficult-to-treat disease and incomplete therapeutic responses. Although these nosological entities are clinically and cytologically defined, they should not be considered synonymous with molecular endotypes. Their future value may instead lie in a cytology-informed endotyping strategy integrating morphology with local biomarkers, proteomics, transcriptomics, and single-cell approaches. This Review discusses the cellular players and interactions shaping allergic rhinitis, the biological and clinical relevance of overlapping inflammation, and the potential role of nasal cytology as an interface between clinical phenotyping and molecular endotyping.

## 1 Introduction

Allergic rhinitis (AR) is a chronic inflammatory disease of the nasal mucosa characterized by a clinically relevant IgE-mediated response to inhaled allergens and represents one of the most common allergic disorders worldwide (1, 2). Although the classical pathogenic model emphasizes allergen-specific IgE, T-helper type 2 immunity, mast-cell activation, and eosinophilic inflammation, it has become increasingly evident that this framework does not fully account for the biological heterogeneity observed at the nasal mucosal level. Patients sharing the same diagnosis of AR may differ considerably in symptom persistence, inflammatory-cell composition, local tissue responses, and response to treatment.

The nasal mucosa should therefore be regarded not as a passive target of the allergic reaction, but as a dynamic immunological ecosystem in which epithelial, immune, stromal, and vascular cells interact continuously. Recent single-cell and multi-omic analyses have substantially expanded this concept by demonstrating aberrant epithelial differentiation, inflammatory activation of fibroblasts, extracellular-matrix remodeling, and enhanced epithelial-stromal communication in AR (3). These findings indicate that the local expression of allergic disease results from coordinated cellular networks extending well beyond a purely eosinophil-centered interpretation of type 2 inflammation.

This local complexity also raises an important distinction between allergic sensitization and mucosal inflammatory expression. Demonstration of allergen-specific IgE identifies an immunological mechanism, but does not necessarily define the entire cellular landscape of the target organ. Nasal cytology provides direct access to this local compartment by allowing the identification of epithelial cells and inflammatory populations, including eosinophils, mast cells, neutrophils, and lymphocytes, as well as their coexistence, activation, and degranulation states (4). Importantly, the cytological expression of AR is not uniform and may vary according to allergen exposure, season, disease activity, treatment, and concomitant inflammatory mechanisms.

This issue becomes especially important in patients in whom established AR coexists with recognized non-allergic cellular rhinitis entities. Early clinical-cytological observations showed that a subgroup of patients with pollen-induced AR continued to experience symptoms outside the expected allergen-exposure period and displayed persistent eosinophilic and/or mast-cell inflammation (5). Subsequent studies extended this observation and led to the concept of overlapping rhinitis, in which an allergic mechanism coexists in the same patient with non-allergic rhinitis with eosinophils (NARES), non-allergic rhinitis with mast cells (NARMA), or non-allergic rhinitis with eosinophils and mast cells (NARESMA) (6). In patients with house dust mite-induced AR, for example, nasal cytology identified concomitant non-allergic cellular rhinitis in a substantial proportion of subjects, further supporting the concept that an allergic diagnosis does not necessarily correspond to a single local inflammatory expression.

These observations challenge a strictly binary separation between allergic and non-allergic rhinitis and support a multidimensional interpretation in which allergen sensitization and local cellular inflammation represent related but distinct biological dimensions. Clinically and cytologically defined entities may represent the morphological expression of distinct biological programs whose molecular signatures remain to be fully characterized. In this perspective, nasal cytology may provide an accessible morphological readout of mucosal inflammatory architecture that can be integrated with local biomarkers, proteomics, transcriptomics, and single-cell approaches.

This Review examines AR as a dynamic mucosal cellular ecosystem, with particular emphasis on mast cell-eosinophil interactions, the biological and clinical significance of overlapping rhinitis, and the potential role of nasal cytology as an interface between clinical phenotyping and molecular endotyping.

## 2 The nasal mucosa as an immunological ecosystem

The nasal mucosa represents the first structural and immunological interface between the respiratory tract and the external environment. Its function extends well beyond that of a physical barrier, as epithelial, stromal, vascular, neuronal, and immune compartments continuously exchange signals that maintain tissue homeostasis and coordinate responses to allergens, microorganisms, pollutants, and other environmental stimuli. In allergic rhinitis (AR), disruption of this equilibrium transforms the mucosa into an active inflammatory microenvironment in which structural cells participate directly in disease initiation, amplification, and persistence.

### 2.1 The epithelial barrier as an active immune compartment

The respiratory epithelium lining most of the nasal cavity consists predominantly of pseudostratified ciliated columnar epithelium containing ciliated, secretory/goblet, and basal cells. These populations perform complementary functions: ciliated cells sustain mucociliary clearance, secretory cells contribute to mucus production and antimicrobial defense, whereas basal cells act as progenitors responsible for epithelial renewal and repair. Far from being a static cellular layer, the nasal epithelium senses environmental perturbations and communicates with the underlying immune compartment through cytokines, chemokines, alarmins, and other mediators.

Recent evidence has particularly changed the view of basal cells in AR. Single-cell RNA sequencing has demonstrated that basal cells are heterogeneous and substantially altered in allergic disease. Ruysseveldt et al. showed an increased basal-cell fraction in the nasal epithelium of patients with AR, together with loss of potentially protective subsets and enrichment of an allergy-associated proinflammatory population. Importantly, basal cells derived from AR patients also exhibited impaired proliferation, wound healing, and barrier formation, indicating that epithelial progenitor dysfunction may actively contribute to persistence of mucosal barrier abnormalities rather than merely representing a consequence of inflammation (7).

Barrier dysfunction is therefore increasingly regarded as an integral component of AR pathogenesis. Impaired epithelial junctions facilitate penetration of allergens and environmental agents into the subepithelial compartment, while type 2 cytokines and inflammatory mediators can further compromise epithelial integrity, establishing a self-amplifying loop between structural damage and immune activation. Recent tissue and experimental data provide additional support for this concept. Increased epithelial expression of S100A8 has been demonstrated in AR and was associated with reduced expression of the tight-junction proteins ZO-1, E-cadherin, and occludin. In cultured human nasal epithelial cells, house dust mite exposure induced S100A8 and disrupted tight-junction protein expression, whereas S100A8 silencing partially restored barrier-associated proteins (8). These findings exemplify how epithelial-derived molecular programs may translate environmental exposure into persistent mucosal dysfunction.

### 2.2 Beyond the epithelium: stromal and intercellular networks

The concept of the nasal mucosa as an integrated ecosystem has been strengthened considerably by recent single-cell multi-omic analyses. In a comprehensive atlas including more than one million nasal mucosal cells, Liu et al. identified marked alterations not only within epithelial populations but also in stromal compartments. AR was associated with impaired maturation of basal and club cells, inflammatory fibroblast activation, extracellular-matrix remodeling, and enhanced epithelial-stromal cross-talk, together with cell subset-specific transcriptional and epigenetic abnormalities (3).

Such findings shift the biological interpretation of AR from an inflammatory infiltrate superimposed on otherwise passive tissue toward a model of coordinated tissue dysfunction. Fibroblasts, endothelial cells, epithelial progenitors, differentiated epithelial cells, and resident or recruited immune populations participate in interconnected signaling networks. The resulting mucosal phenotype therefore reflects not simply the presence or absence of a particular inflammatory cell, but the cumulative output of cell recruitment, activation, survival, degranulation, tissue repair, vascular responses, and remodeling.

Single-cell studies of patients with concomitant AR and asthma similarly identified structural abnormalities of ciliated cells and epithelial barrier pathways, together with increased basal-cell proliferation and altered communication between epithelial and immune populations, further supporting the concept that epithelial remodeling and immune inflammation are biologically intertwined (9). These observations also reinforce the relevance of the nasal compartment as an accessible site in which airway inflammatory biology can be studied at both cellular and molecular levels.

From this perspective, the morphological appearance of the nasal mucosa acquires biological significance. Histology captures tissue architecture, epithelial integrity, subepithelial infiltration, vascular and stromal changes, whereas nasal cytology provides a complementary view of the cellular populations present at the mucosal surface and their activation state. Representative cytological and histological images of normal and pathological nasal mucosa are shown in **Figure 1**, illustrating the transition from the surface cellular readout to the broader tissue architecture and inflammatory context. These approaches interrogate different levels of the same biological system. The transition from normal mucosal architecture to allergic inflammation is therefore accompanied not only by quantitative changes in inflammatory cells, but by a broader reorganization of epithelial, stromal, and immune relationships.

**Figure 1:**
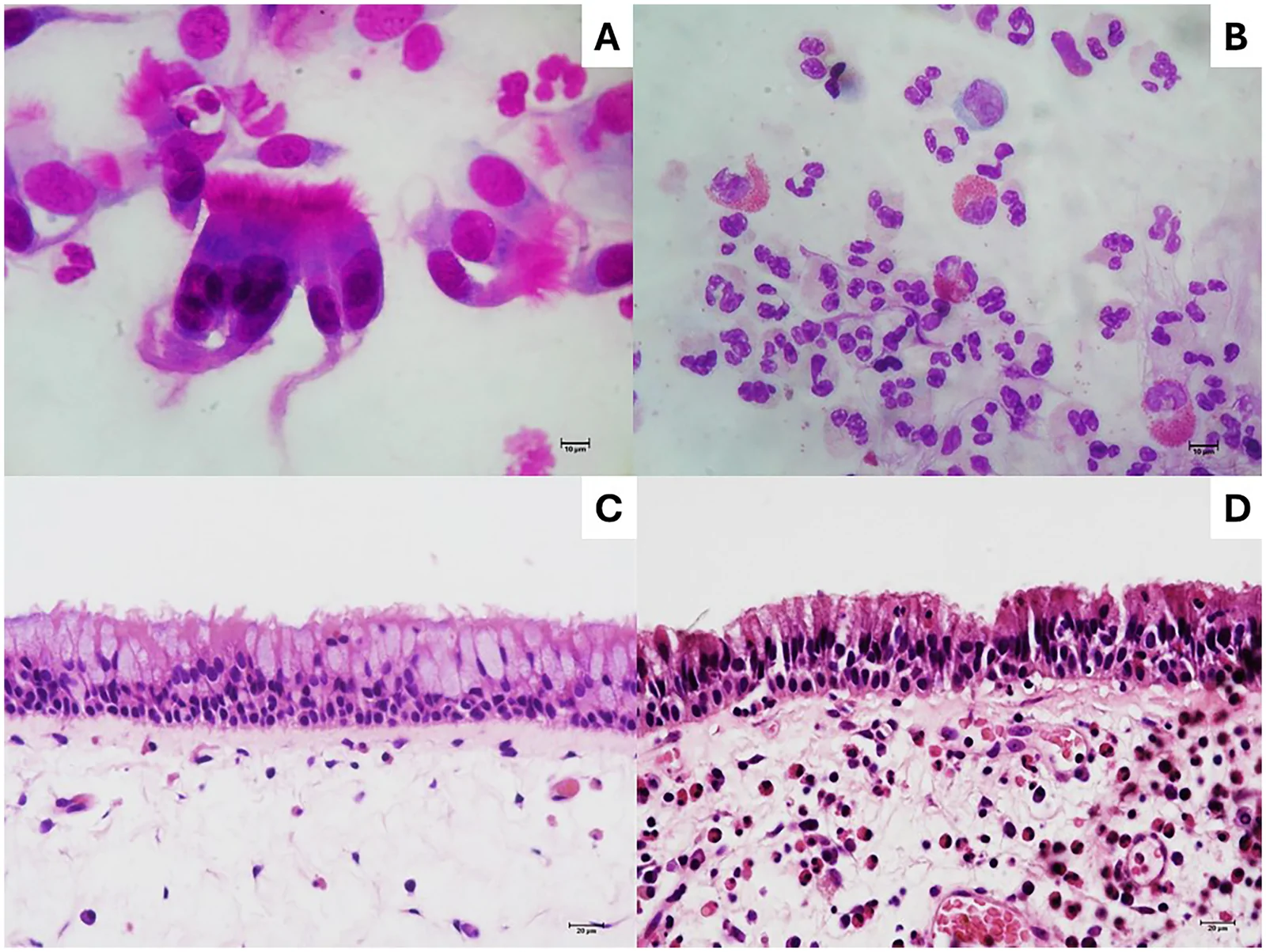
Cytological and histological views of normal and pathological nasal mucosa. **(A)** Normal nasal cytology showing numerous ciliated epithelial cells and only rare neutrophils. **(B)** Pathological nasal cytology showing a mixed inflammatory infiltrate characterized by numerous neutrophils, eosinophils, and lymphocytes. Both cytological specimens were stained with May–Grünwald–Giemsa (MGG) and examined at ×1000 magnification. **(C)** Histology of normal nasal mucosa showing a compact ciliated epithelium and a lamina propria without evident inflammatory infiltration. **(D)** Histology of pathological nasal mucosa showing a partially discontinuous epithelium and evident inflammatory-cell infiltration involving both the epithelium and the lamina propria. Histological sections were stained with hematoxylin–eosin (H&E) and examined at ×400 magnification. Original microscopy images are from the author’s archive.

This integrated view provides the biological foundation for understanding why apparently similar clinical forms of AR may display different local cellular landscapes and prepares the ground for examining the epithelial signals and innate and adaptive immune pathways that drive type 2 amplification.

## 3 Epithelial alarmins, ILC2s, and type 2 amplification

The epithelial barrier does not merely separate the external environment from the underlying immune compartment; it actively senses allergens, pollutants, pathogens, and tissue injury and converts these signals into immune activation. Among the mediators released by activated or damaged epithelial cells, thymic stromal lymphopoietin (TSLP), interleukin (IL)-25, and IL-33 have emerged as major upstream regulators of type 2 inflammation in the upper airway. By acting on both innate and adaptive immune populations, these epithelial-derived alarmins provide a mechanistic bridge between barrier perturbation and the recruitment and activation of inflammatory effector cells.

Type 2 innate lymphoid cells (ILC2s) occupy a central position within this axis. Unlike conventional T lymphocytes, ILC2s do not require antigen-specific receptor recognition and can respond rapidly to epithelial-derived signals at mucosal surfaces. In allergic rhinitis (AR), increased expression of IL-25, IL-33, and TSLP has been reported in the nasal compartment, and these mediators promote ILC2 activation, proliferation, and cytokine production (10). Activated ILC2s predominantly release IL-5 and IL-13, thereby promoting eosinophil recruitment and survival, mucus production, goblet-cell responses, and epithelial remodeling. This rapid innate pathway operates in parallel with, and interacts extensively with, conventional allergen-specific Th2 immunity.

### 3.1 ILC2s as amplifiers of mucosal type 2 inflammation

ILC2 biology has substantially modified the classical interpretation of allergic inflammation. The conventional model of AR emphasized allergen presentation, Th2 differentiation, IgE production, mast-cell sensitization, and subsequent eosinophilic recruitment. The recognition of ILC2s has introduced an additional, antigen-independent amplification pathway in which epithelial stress can rapidly generate type 2 cytokine responses before, or alongside, conventional adaptive immune activation.

ILC2s are particularly enriched at mucosal barrier sites and are strategically positioned beneath the nasal epithelium, where they can respond directly to local environmental signals. Their activation is not restricted to epithelial cytokines. Lipid mediators, including prostaglandin D2 and cysteinyl leukotrienes, as well as neuropeptides and other tissue-derived signals, can further enhance ILC2 migration and cytokine production (10). This broader responsiveness illustrates how allergic inflammation can be modulated by neural, epithelial, stromal, and metabolic inputs, reinforcing the concept of AR as a network disease rather than a linear immunological cascade.

Recent reviews of nasal mucosal immunity have further emphasized the plasticity of ILC populations and the existence of locally resident progenitors capable of adapting to the surrounding cytokine milieu. ILC2s are characterized by GATA3-dependent transcriptional programs and, following stimulation by IL-25, IL-33, and TSLP, produce large amounts of IL-5 and IL-13. These cytokines favor eosinophilic inflammation, mucus hypersecretion, epithelial remodeling, and persistent type 2 immune activity (11). Although much of the detailed evidence on tissue remodeling derives from chronic rhinosinusitis with nasal polyps, the same epithelial-ILC2 signaling axis is relevant to severe AR and provides an important mechanistic framework for understanding local type 2 amplification.

### 3.2 Crosstalk between innate and adaptive immunity

ILC2s and Th2 cells should not be viewed as independent sources of the same cytokines. Instead, they participate in reciprocal regulatory networks. ILC2-derived cytokines can influence dendritic-cell activity and favor Th2 polarization, whereas activated Th2 cells provide additional IL-4, IL-5, and IL-13 that reinforce the local type 2 environment. Conversely, dendritic cells and regulatory T cells can modulate ILC2 activity, creating a dynamic balance between amplification and restraint of mucosal inflammation (10).

This interaction is particularly relevant to the interpretation of nasal cellular phenotypes. IL-5 promotes eosinophil differentiation, recruitment, survival, and activation, whereas IL-13 contributes to epithelial dysfunction, mucus hypersecretion, and tissue remodeling. Mast cells, through IgE-dependent activation and the release of histamine, proteases, prostaglandin D2, cysteinyl leukotrienes, and cytokines, can further reinforce ILC2 and eosinophil responses. Thus, the eosinophil-rich or eosinophil-mast-cell-rich cytological picture observed in some patients should be regarded as the visible downstream expression of a broader epithelial-innate-adaptive immune network rather than as an isolated cellular event (**Figure 2**).

**Figure 2:**
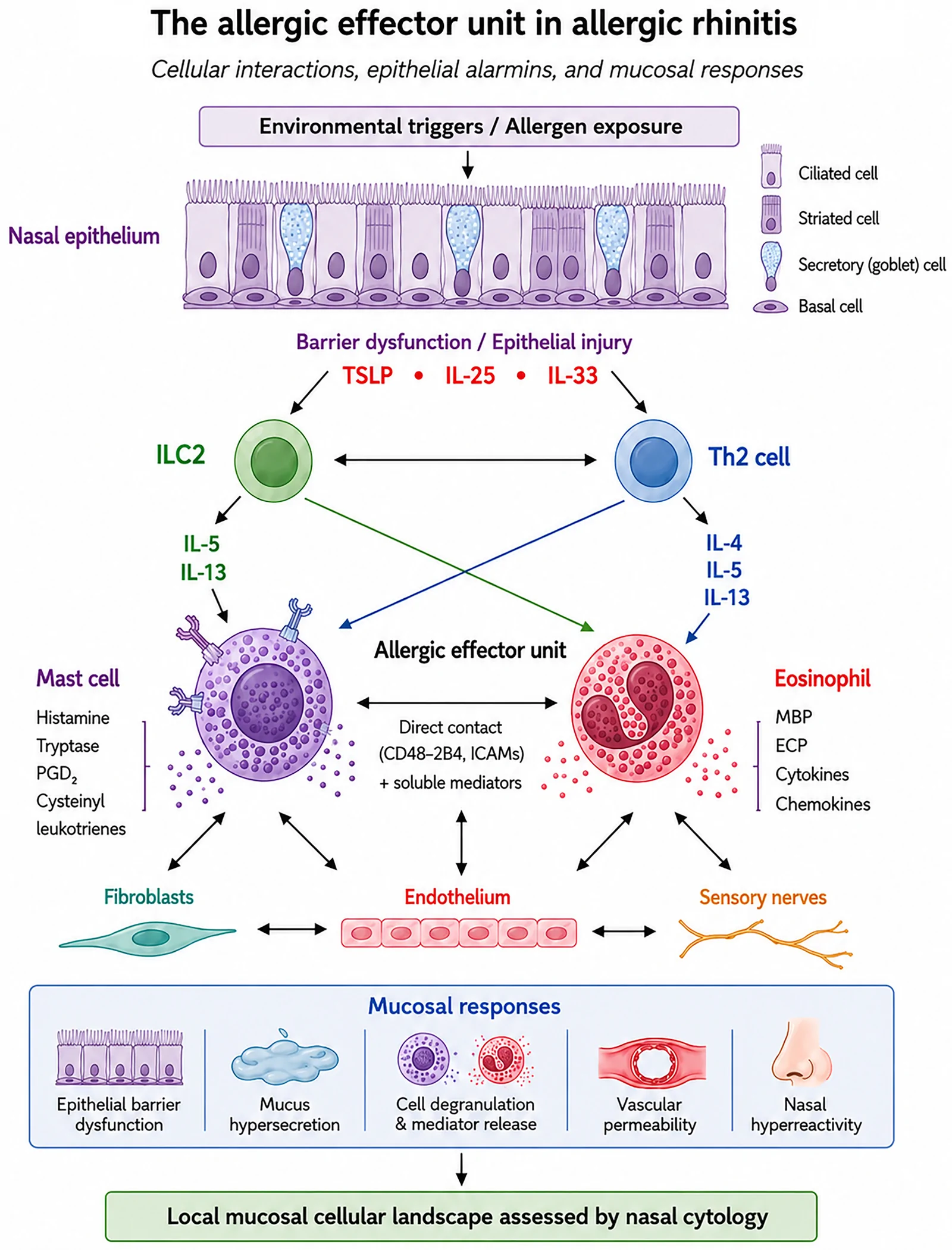
The mucosal cellular ecosystem of allergic rhinitis. Conceptual schematic. Environmental triggers and allergen exposure interact with the nasal epithelium, promoting barrier dysfunction and release of epithelial alarmins, including TSLP, IL-25, and IL-33. These signals activate and amplify type 2 immune pathways involving ILC2 and Th2 cells, which in turn promote mast-cell and eosinophil recruitment and activation. Mast cells and eosinophils communicate through soluble mediators and direct physical interactions, including CD48-2B4-dependent contact, within the allergic effector unit. Fibroblasts, endothelial cells, and sensory nerves further contribute to the local inflammatory network. The resulting mucosal responses include epithelial dysfunction, mucus hypersecretion, degranulation and mediator release, increased vascular permeability, and nasal hyperreactivity. Nasal cytology provides a clinically accessible readout of the resulting local cellular landscape.

Similar cytological appearances can arise through different upstream mechanisms. An eosinophilic infiltrate may reflect classical allergen-specific IgE/Th2 activation, epithelial alarmin-driven ILC2 responses, or convergence of both pathways. Likewise, mast cells and eosinophils in the same cytogram may represent coordinated activation within a broader type 2 network. Cytology therefore captures the downstream cellular expression of inflammation, but not the full mechanism that generated it.

This distinction underpins the approach developed in this Review: nasal cytology identifies the inflammatory cells present at the target organ and their activation or degranulation state, whereas molecular and functional methods are needed to resolve the upstream programs sustaining those patterns. The next section focuses on mast cells and eosinophils as interacting components of this mucosal network.

## 4 Mast cells and eosinophils: from individual effectors to an interacting cellular network

Mast cells and eosinophils are among the principal effector cells of type 2 inflammation, but their contribution to allergic rhinitis (AR) extends beyond their individual functions. Both populations occupy strategic positions within the nasal mucosa, respond to overlapping sets of cytokines, chemokines, lipid mediators, and tissue-derived signals, and can reciprocally influence recruitment, activation, survival, and mediator release. Their coexistence should therefore be interpreted within an interactive cellular network rather than as the simple simultaneous presence of two independent inflammatory populations.

### 4.1 Mast cells: early effectors and amplifiers of mucosal inflammation

Mast cells are tissue-resident immune cells concentrated at host-environment interfaces, including the respiratory mucosa. In sensitized individuals, allergen-induced cross-linking of IgE bound to FcϵRI triggers rapid degranulation and release of preformed mediators such as histamine and proteases, followed by the synthesis of prostaglandins, cysteinyl leukotrienes, cytokines, and chemokines. These mediators account for major components of the immediate allergic response, including sneezing, itching, rhinorrhea, vascular permeability, and mucosal edema, but mast-cell activity extends well beyond this early phase (12).

Mast cells can influence vascular, epithelial, neuronal, innate, and adaptive immune compartments. Their close relationship with sensory nerve endings is particularly relevant because reciprocal mast cell-neuronal signaling may amplify itch, sneezing, secretion, and mucosal hyperreactivity and contribute to positive-feedback neuroimmune circuits in allergic inflammation (13).

### 4.2 Eosinophils: tissue injury, persistence, and remodeling

Eosinophils are recruited into the nasal mucosa through a coordinated process involving IL-5, eotaxins, adhesion molecules, and local chemotactic signals. Once activated, they release granule proteins, including major basic protein and eosinophil cationic protein, together with cytokines, lipid mediators, and other inflammatory molecules. These products can contribute to epithelial injury, altered barrier function, mucus secretion, and persistence of local inflammation.

The biological significance of eosinophils therefore depends not only on their absolute number, but also on their activation and degranulation state. For nasal cytology, this matters because free eosinophilic granules and morphological signs of degranulation may reveal active tissue inflammation that a simple cell count would not adequately represent.

### 4.3 Mast cell-eosinophil crosstalk and the allergic effector unit

Experimental and translational evidence supports bidirectional communication between mast cells and eosinophils. This interaction has been conceptualized as the allergic effector unit, in which the two populations communicate through soluble mediators as well as through direct physical cell-cell contacts (14). The interaction involves cytokines, chemokines, lipid mediators, granular products, and surface receptor-ligand systems, with the CD48-2B4 axis emerging as one of the best-characterized contact-dependent pathways.

Importantly, mast cells and eosinophils do not merely co-localize within allergic tissues. Elishmereni et al. demonstrated that the two cell types can form stable conjugates with clear membrane contact and that mast-cell-dependent enhancement of eosinophil survival was strongly dependent on physical cell-cell interaction (15). A subsequent study showed that eosinophils enhanced basal mast-cell mediator release and co-stimulated IgE-activated mast cells through physical contact involving CD48-2B4 interactions, while mast cells reciprocally promoted eosinophil migration and activation through paracrine mechanisms (16).

From a morphological perspective, these findings are especially informative. Nasal cytology may occasionally show mast cells and eosinophils in very close apposition (**Figure 3**). This finding is morphologically compatible with the direct physical contacts described experimentally within the allergic effector unit, while cytology alone cannot establish the functional nature of the interaction.

**Figure 3:**
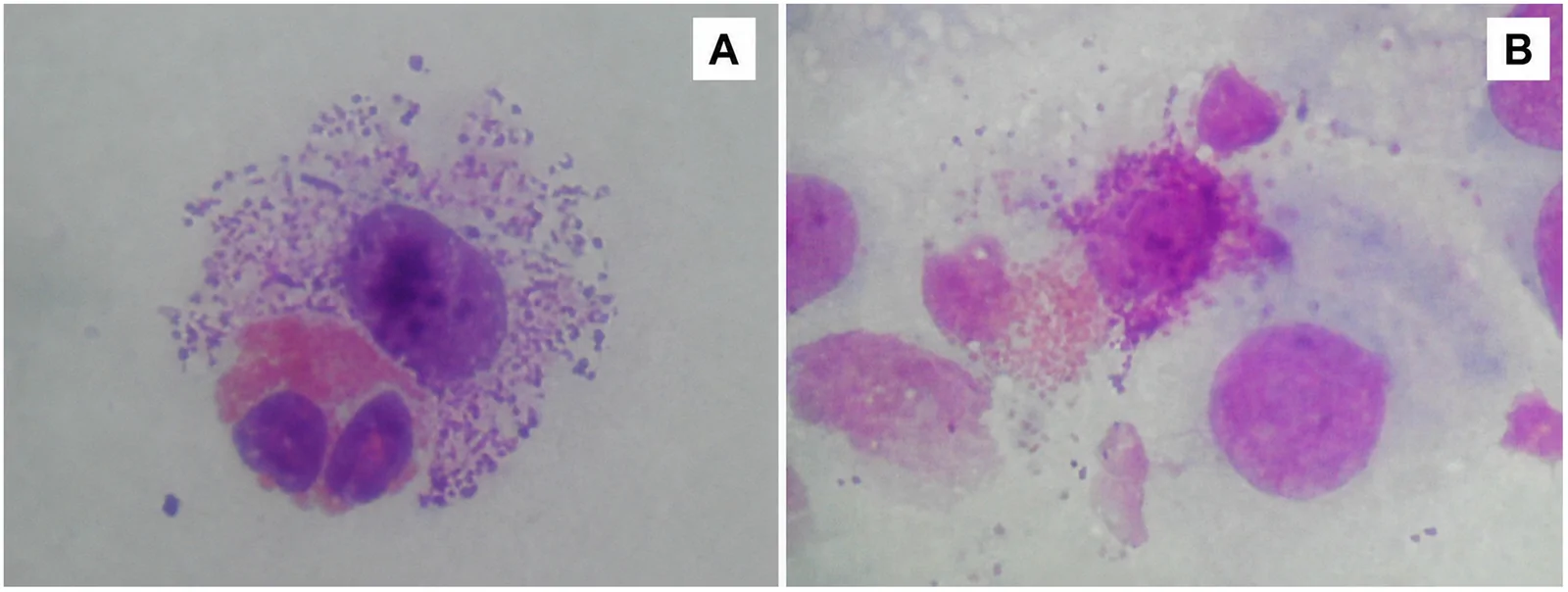
Mast cell–eosinophil proximity in nasal cytology. **(A)** Representative nasal cytology showing very close apposition between a mast cell and an eosinophil, morphologically compatible with the stable cell conjugates and direct physical contacts described experimentally within the mast cell–eosinophil allergic effector unit. **(B)** Representative cytological field showing concomitant mast-cell and eosinophil degranulation, illustrating the coexistence of activated effector-cell populations. Nasal cytology specimens were stained with May–Grünwald–Giemsa (MGG) and examined at an initial microscopic magnification of ×1000, with an additional camera magnification factor (CMF) of ×2. Cytological morphology documents cellular identity, proximity, and degranulation, but does not by itself demonstrate functional signaling or causal crosstalk. The two panels are original micrographs placed side by side; no feature-level image modification was performed. Original microscopy images are from the author’s archive.

The biological significance of this interaction becomes especially relevant when considering non-allergic rhinitis with eosinophils and mast cells (NARESMA). In its original prospective characterization, NARESMA was identified as a distinct nosological entity defined by the concomitant presence of eosinophils and mast cells and associated with a more severe clinical profile than other cellular forms of non-allergic rhinitis (17).

At present, NARESMA should not be equated mechanistically with the allergic effector unit. However, the coexistence of mast cells and eosinophils in a recognized clinical-cytological entity, together with experimental evidence of reciprocal physical and paracrine interaction, raises the biologically relevant hypothesis that eosinophil-mast-cell-rich states may reflect coordinated cellular programs rather than simple additive inflammation. Whether NARESMA corresponds to a reproducible molecular signature specifically associated with enhanced mast cell-eosinophil crosstalk remains an important question for future investigation.

### 4.4 From cellular activation to nasal hyperreactivity

The functional consequences of eosinophil and mast-cell activation may also be clinically observable. In a study including 671 subjects undergoing nasal endoscopy, endoscopy-induced nasal hyperreactivity, expressed as a volley of sneezes, was markedly more frequent in cellular non-allergic rhinitis than in AR, occurring in 83% of patients with NARES, 70.6% with NARMA, and 76% with NARESMA, compared with 19% of those with AR (18).

The same study proposed that degranulation-associated epithelial damage and subsequent exposure or sensitization of mucosal sensory pathways could contribute to this enhanced reactivity. These findings should not be interpreted as defining universal cellular thresholds for nasal symptoms, but they provide a clinically relevant example of how local cellular composition and activation may translate into functional mucosal behavior.

Viewed together, these data argue against regarding mast cells and eosinophils merely as histological or cytological markers of type 2 inflammation. Their recruitment, activation, degranulation, reciprocal signaling, and interaction with epithelial, vascular, and neuronal compartments contribute to the dynamic organization of the nasal inflammatory microenvironment. Nasal cytology can visualize the cellular output of these interactions, whereas molecular approaches are required to determine the signaling programs that sustain them. This distinction provides the basis for examining why local mucosal inflammation may not be faithfully reflected by systemic biomarkers.

## 5 Local versus systemic inflammation and the heterogeneity of allergic rhinitis

Allergic rhinitis is commonly diagnosed through the combination of compatible symptoms and evidence of allergen sensitization, usually obtained by skin prick testing or serum-specific IgE. However, these systemic markers do not necessarily reproduce the biological state of the nasal mucosa. The target organ represents a distinct inflammatory compartment in which immune-cell recruitment, activation, mediator release, epithelial dysfunction, and tissue remodeling are regulated locally. Consequently, systemic sensitization and local mucosal inflammation should be considered related but non-equivalent dimensions of allergic disease.

### 5.1 The target organ may diverge from systemic biomarkers

The distinction between systemic and local immunity is particularly evident in local allergic rhinitis (LAR), a condition characterized by symptoms suggestive of allergic rhinitis despite negative conventional systemic allergy tests. Current evidence indicates that LAR is associated with a localized type 2 response, and nasal allergen challenge remains the reference diagnostic procedure for demonstrating allergen-specific nasal reactivity (19, 20). Local specific IgE, basophil activation testing, and nasal cytology may provide complementary information, but none of these approaches alone can substitute for demonstration of clinically relevant local allergen reactivity (19, 21).

The interpretation of local specific IgE illustrates this problem particularly well. In a study evaluating nasal specific IgE in subjects with allergic rhinitis, non-allergic rhinitis, and healthy controls, local IgE could also be detected in a proportion of non-allergic subjects and even in healthy individuals, suggesting that the mere presence of nasal allergen-specific IgE does not necessarily identify a clinically meaningful local allergic response (22). Accordingly, local biomarkers should be interpreted within the appropriate clinical and functional context rather than considered diagnostic in isolation.

The same caution applies to nasal cytology. Eosinophilic or mast-cell inflammation documents the presence of a local inflammatory response, but it does not by itself establish whether that response is driven by systemic allergy, local allergy, non-allergic cellular rhinitis, or the coexistence of more than one mechanism. Thus, local cellular inflammation identifies what is occurring in the target organ, whereas allergological testing and functional challenge help define the mechanism responsible for it.

### 5.2 Allergic rhinitis does not correspond to a single cellular profile

Even in patients with unequivocally established systemic AR, the cytological pattern is not uniform. The inflammatory composition of the nasal mucosa varies according to the type of allergen, intensity and duration of exposure, season, disease activity, and potentially treatment.

In a cohort of monosensitized patients, significant differences in nasal cytology were observed according to allergen type. Pollen-sensitized subjects showed higher concentrations of eosinophils, degranulated eosinophils, mast cells, and degranulated mast cells, whereas house dust mite-sensitized patients showed a relatively greater representation of goblet cells. Nasal airflow resistance also varied according to the sensitizing allergen (23). These findings support the concept that the same clinical diagnosis of AR may correspond to different local inflammatory configurations.

Disease severity may also influence the cellular landscape. In grass pollen-induced AR, patients with moderate-to-severe disease showed significantly higher mast-cell and lymphocyte/plasma-cell counts than patients with mild disease, whereas the distinction between intermittent and persistent AR was less clearly reflected by cytology (24). This suggests that the complexity of the inflammatory infiltrate, rather than the abundance of a single cell type, may better reflect the biological intensity of mucosal disease.

### 5.3 The nasal cellular phenotype is dynamic

The cytological phenotype of AR should not be considered a fixed characteristic of the patient. Longitudinal observations demonstrate that the cellular composition of the nasal mucosa may change substantially over time in relation to allergen exposure.

This is particularly evident in pollen-induced AR, where eosinophils, mast cells, and other inflammatory populations increase during periods of allergen exposure and decrease outside the relevant pollen season. Similar temporal variability has also been demonstrated in house dust mite allergy, traditionally considered a perennial disease. In a longitudinal study in which patients monosensitized to house dust mites underwent monthly nasal cytology for one year, neutrophils represented the predominant population, while the period from October to April was characterized by increased neutrophils together with significant numbers of eosinophils, mast cells, and lymphocytes/plasma cells (25). The authors related these changes to seasonal fluctuations in environmental mite allergen load.

The longitudinal data emphasize that a cytological phenotype is a time-dependent readout of the nasal mucosa rather than an immutable patient trait. Its interpretation therefore requires knowledge of allergen exposure, season, treatment status, and clinical activity.

This temporal dimension becomes particularly useful when distinguishing isolated AR from overlapping rhinitis. In seasonal disease, marked eosinophilic and/or mast-cell inflammation outside the relevant exposure period is not readily explained by the allergic mechanism alone; in perennial allergy, the expected background of minimal persistent inflammation must instead be taken into account.

Allergic sensitization, therefore, does not define a unique nasal cellular phenotype. The local picture is shaped by allergen-specific immunity, exposure, tissue responses, and concomitant inflammatory mechanisms, providing the conceptual transition to overlapping rhinitis.

## 6 Beyond the allergic label: overlapping rhinitis

Allergic rhinitis and non-allergic cellular rhinitis should not necessarily be regarded as mutually exclusive conditions. A patient with documented allergen sensitization may simultaneously express an additional cellular inflammatory disorder of the nasal mucosa, giving rise to the concept of overlapping rhinitis (OR) (5, 6). In this setting, the allergic component identifies an allergen-specific immune mechanism, whereas nasal cytology may reveal a concomitant inflammatory phenotype that cannot be fully explained by the expected allergic response alone.

Clinically, this matters because an allergic diagnosis may otherwise be assumed to explain every symptom and inflammatory finding. Persistent symptoms beyond the expected exposure period, marked non-specific hyperreactivity, incomplete therapeutic response, or a cytological pattern disproportionate to the expected allergic inflammation should prompt consideration of an additional cellular rhinitis.

Overlapping rhinitis can therefore be viewed as the coexistence, in the same patient, of an allergen-specific mechanism and a concomitant non-allergic cellular inflammatory disorder. These components may interact, but they are not interchangeable. In this Review, this clinical-cytological construct is distinct from mixed rhinitis, a broader clinical coexistence of allergic and non-allergic triggers or mechanisms, and from local allergic rhinitis, which is defined by local allergen-specific reactivity in the absence of systemic sensitization.

### 6.1 Origins of the overlapping rhinitis concept

The concept of overlapping rhinitis emerged from the observation that a proportion of patients with established allergic rhinitis displayed clinical and cytological features that could not be adequately explained by allergen sensitization alone. In an early study of 519 patients with pollen-induced allergic rhinitis, 60 subjects (11.5%) showed an atypical clinical course characterized by persistent or recurrent symptoms outside the expected pollen season, non-specific nasal hyperreactivity, and cytological evidence of persistent eosinophilic and/or mast-cell inflammation (5). These patients also showed a higher prevalence of associated conditions such as asthma and nasal polyposis.

These observations suggested that allergic sensitization and non-allergic cellular inflammation may coexist in the same individual. The subsequent definition of overlapping rhinitis emphasized that the presence of an allergic mechanism does not exclude an additional cellular rhinitis such as NARES, NARMA, or NARESMA (6). The available evidence is mainly observational and should be interpreted in light of patient selection, timing of sampling, treatment, and allergen exposure; prospective multicenter studies are needed to define the reproducibility and clinical significance of these patterns.

However, an important distinction is required. The finding of eosinophils or mast cells in an allergic patient is not sufficient, by itself, to diagnose overlapping rhinitis, because these cells may be part of the expected allergen-driven inflammatory response. The diagnosis therefore depends on demonstrating a cellular pattern whose intensity, persistence, or temporal distribution is not adequately explained by the patient’s allergic sensitization and current allergen exposure.

### 6.2 Allergen exposure patterns define different cytological contexts

Although the ARIA classification, based on symptom duration and severity, remains the current clinical framework for allergic rhinitis, the distinction between seasonal and perennial allergen exposure retains specific value for cytological interpretation. In the present context, these terms are therefore not used as an alternative clinical classification of AR, but as exposure-based categories reflecting different patterns of mucosal inflammatory response. Pollen allergens typically produce temporally restricted exposure, whereas house-dust mites and animal-derived allergens are associated with more continuous exposure and different patterns of persistent mucosal inflammation. This distinction is particularly relevant when nasal cytology is used to identify an inflammatory pattern that is discordant with that expected from the patient’s allergen exposure. The interpretation of nasal cytology in suspected overlapping rhinitis depends critically on the type and timing of allergen exposure.

In seasonal allergic rhinitis, the most informative assessment is often obtained outside the relevant pollen season. During active allergen exposure, the presence of eosinophils and mast cells may simply represent the expected local allergic response. Conversely, persistence of significant eosinophilic and/or mast-cell inflammation when the corresponding pollen is absent or clinically irrelevant raises the possibility of an associated cellular rhinitis.

Accordingly, in a patient sensitized, for example, to a seasonal pollen allergen, an out-of-season rhinocytogram showing persistent eosinophilia may support the coexistence of NARES, predominant mast-cell inflammation may suggest NARMA, and a combined eosinophil-mast-cell pattern may indicate NARESMA, provided that the findings are consistent with the clinical history.

The situation is different in perennial allergic rhinitis, particularly in patients sensitized to house-dust mites. In these subjects, the nasal mucosa may display a state of minimal persistent inflammation, even in the absence of marked symptoms, characterized predominantly by neutrophilic inflammation with limited eosinophilic representation and occasional cellular degranulation (23–25). Therefore, the mere presence of some eosinophils does not necessarily indicate an overlapping disorder.

What becomes diagnostically informative is a cytological pattern that clearly exceeds this expected background. A disproportionately intense eosinophilic infiltrate, marked mast-cell presence or degranulation, or the simultaneous prominence of both cell populations may indicate the coexistence of an additional cellular rhinitis.

Observations in patients with house-dust-mite allergic rhinitis support this interpretation. In a multicenter study, a substantial proportion of patients with mite allergy showed cytological features consistent with an associated non-allergic cellular rhinitis, with NARESMA representing the most frequent pattern among the overlapping phenotypes (26). These findings reinforce the need to interpret perennial allergic inflammation against its expected cytological background rather than applying the same criteria used for seasonal disease.

### 6.3 Cytological recognition of overlapping rhinitis

Cytological recognition of overlapping rhinitis requires the rhinocytogram to be interpreted together with the clinical, allergological, and temporal context.

Operationally, the observed cellular pattern should be judged against the exposure-specific background described above. In seasonal AR, persistent eosinophilic and/or mast-cell inflammation outside the relevant pollen period supports an additional cellular rhinitis; in perennial AR, particularly house-dust-mite allergy, the key clue is eosinophilic or mast-cell inflammation that clearly exceeds minimal persistent inflammation. In neither setting is the cell pattern diagnostic in isolation.

A practical synthesis of the main clinical and cytological clues that should raise suspicion of overlapping rhinitis is provided in **Table 1**.

**Table 1:** Clinical and cytological clues suggesting overlapping rhinitis.

Domain	Findings suggesting possible overlapping rhinitis
Clinical history	Persistent or recurrent nasal symptoms not fully explained by the expected allergen exposure
Non-specific hyperreactivity	Symptoms triggered by temperature changes, strong odors, smoke, chlorine, or other irritants
Temporal discordance	Persistence of symptoms outside the relevant pollen season in seasonal allergic rhinitis
Olfactory/gustatory symptoms	Hyposmia, anosmia, or taste disturbances disproportionate to the expected allergic course
Associated clinical background	Asthma, nasal polyposis, aspirin/NSAID sensitivity, previous diagnosis of NARES, NARMA, or NARESMA, or longstanding vasomotor symptoms
Medication history	Recurrent or excessive use of topical nasal decongestants
Therapeutic discordance	Incomplete clinical benefit despite appropriate allergen-specific treatment or previous turbinate surgery
Seasonal AR cytology	Persistent or marked eosinophilic and/or mast-cell inflammation outside the corresponding pollen season
Perennial AR cytology	Eosinophilic and/or mast-cell inflammation clearly exceeding the expected minimal persistent inflammatory pattern
Integrated interpretation	Concordance between persistent symptoms, exposure-independent inflammation, and a cytological pattern compatible with NARES, NARMA, or NARESMA

Historical cytological thresholds proposed for the recognition of persistent eosinophilic or mast-cell inflammation may provide operational guidance, but they should not be interpreted as universally validated molecular endotype cut-offs. Their diagnostic meaning depends on the clinical setting, the timing of sampling, and the expected inflammatory pattern associated with the relevant allergen.

No single clinical or cytological finding is sufficient in isolation. Recognition of overlapping rhinitis relies on the concordance between allergen sensitization, allergen exposure, symptom timing, and the local cellular inflammatory profile.

This integrative interpretation is summarized in **Figure 4**.

**Figure 4:**
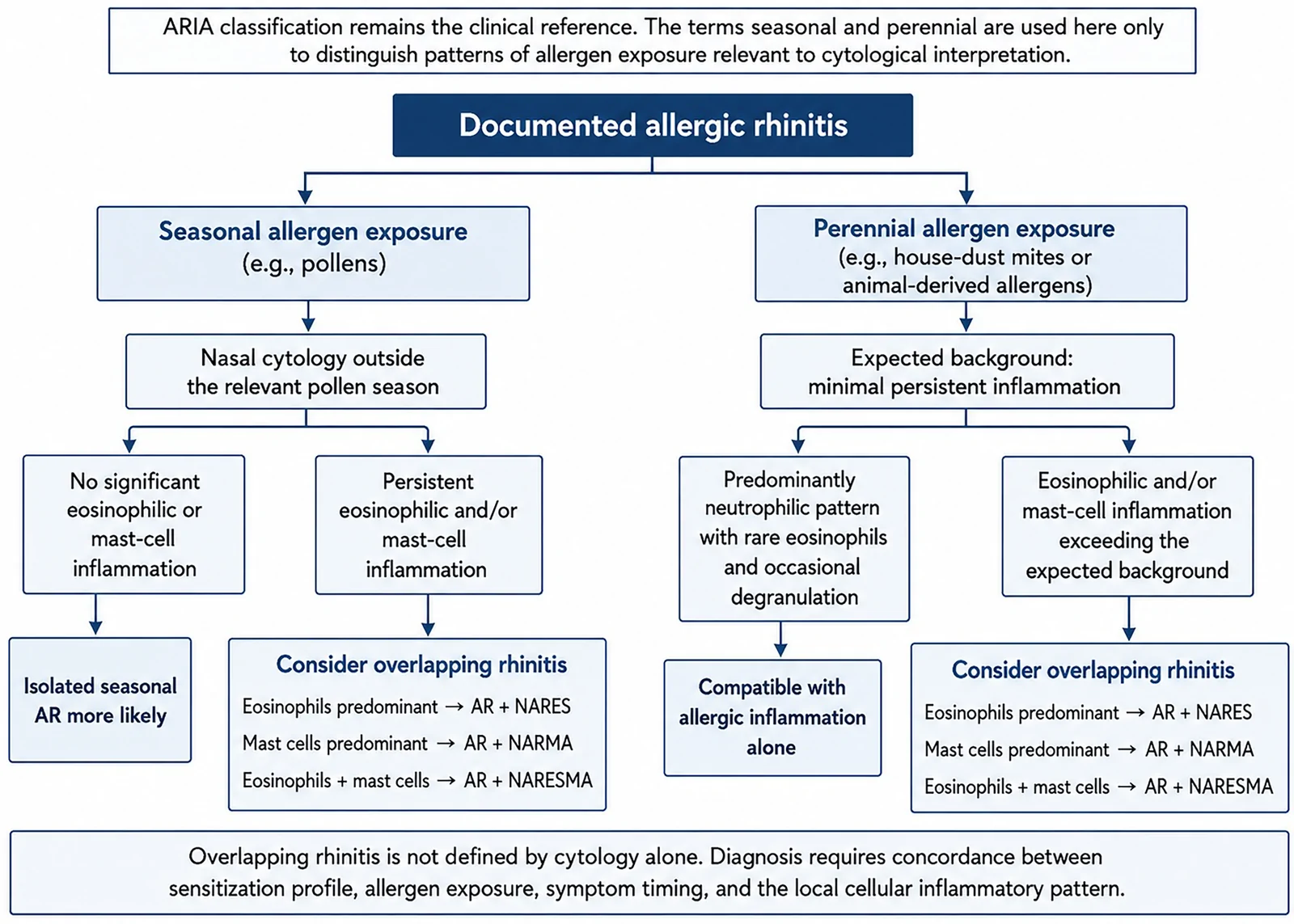
Cytology-informed recognition of overlapping rhinitis. Conceptual flowchart. The figure illustrates how nasal cytology should be interpreted within the clinical, allergological, and temporal context when overlapping rhinitis is suspected. Although the ARIA classification remains the clinical reference for allergic rhinitis, the distinction between seasonal and perennial allergen exposure is retained here exclusively for cytological interpretation, because different exposure patterns are associated with different expected mucosal inflammatory profiles. In seasonal allergen exposure, cytology performed outside the relevant pollen season is particularly informative, since persistent eosinophilic and/or mast-cell inflammation may suggest concomitant NARES, NARMA, or NARESMA. In perennial allergen exposure, particularly house-dust-mite allergy, interpretation should take into account the expected minimal persistent inflammation; eosinophilic and/or mast-cell inflammation clearly exceeding this background may indicate an associated cellular rhinitis. Overlapping rhinitis is therefore not defined by cytology alone, but by the concordance between sensitization profile, allergen exposure, symptom timing, and the local cellular inflammatory pattern.

### 6.4 Overlapping rhinitis and difficult-to-treat disease

The presence of overlapping inflammation may help explain why some patients with apparently well-characterized allergic rhinitis remain symptomatic despite appropriate allergen-directed treatment.

A therapeutic intervention targeting the allergic component does not necessarily eliminate a concomitant non-allergic cellular inflammatory disorder. Allergen immunotherapy, for example, specifically addresses allergen-driven immune responses but may not fully account for an associated NARES, NARMA, or NARESMA component.

Similarly, anatomical treatment alone cannot be expected to normalize an underlying inflammatory phenotype. This concept is supported by observations in highly selected patients undergoing turbinate surgery, in whom cytological overlapping patterns were frequently identified (28). Because such cohorts represent patients with difficult-to-treat disease and surgical indications, these findings should not be extrapolated to the general allergic-rhinitis population. They nevertheless illustrate how persistent cellular inflammation may coexist with allergy in clinically complex patients.

Independent literature on mixed rhinitis also recognizes that allergic and non-allergic mechanisms can coexist in the same patient, although that broader clinical construct is not identical to the cytology-defined overlapping rhinitis discussed here (27). This convergence reinforces the need to look beyond sensitization alone when symptoms or local inflammation remain insufficiently explained.

The clinical relevance of OR therefore lies less in establishing a new epidemiological category than in recognizing a possible explanation for symptom persistence, non-specific hyperreactivity, or incomplete therapeutic response in patients whose disease cannot be adequately described by the allergic label alone.

### 6.5 Overlapping rhinitis as a model of mucosal inflammatory heterogeneity

Overlapping rhinitis provides a clinically accessible example of mucosal inflammatory heterogeneity.

Allergic testing and nasal cytology interrogate complementary dimensions of disease: the former identifies an allergen-specific immune response, whereas the latter describes its local cellular expression and can reveal additional inflammatory programs. These dimensions may coincide, diverge, or overlap.

NARES, NARMA, and NARESMA remain clinically and cytologically defined nosological entities rather than established molecular endotypes. Whether they correspond to reproducible biological programs—and whether NARESMA is linked to enhanced mast cell–eosinophil crosstalk—requires molecular and longitudinal validation.

Accordingly, neither the allergic label nor cytology alone fully defines mechanism. Their integration with exposure history, clinical behavior, and molecular data provides the rationale for cytology-informed endotyping.

## 7 Nasal cytology as a bridge toward cytology-informed endotyping

Nasal cytology directly samples the mucosal surface and provides morphological information on epithelial and inflammatory-cell populations. It is minimally invasive, repeatable, and suitable for longitudinal assessment, allowing recognition of ciliated and goblet cells, neutrophils, eosinophils, mast cells, lymphocytes, microorganisms, and signs of activation or degranulation (29).

Its value extends beyond cell counting. By depicting the inflammatory state of the target organ at a specific time point, cytology complements systemic biomarkers that cannot reproduce the local cellular architecture. This is especially useful in AR, where the same allergological diagnosis may coexist with different mucosal cellular configurations.

### 7.1 From morphology to biologically meaningful cellular phenotypes

The diagnostic value of nasal cytology depends on standardized sampling, processing, staining, and interpretation. Methodological studies have established the feasibility of nasal scraping and differential cell assessment in both clinical practice and research, while more recent consensus initiatives have addressed the need for greater standardization across laboratories and operators (29, 30). A recent expert-based Delphi consensus involving 100 members of the Italian Academy of Nasal Cytology established standardized recommendations for sampling, May-Grünwald-Giemsa staining, microscopic assessment, and clinical interpretation (31).

Standardization is particularly relevant to the concept developed in this Review. The recent Delphi consensus provides an important foundation for reproducible clinical and research use; accordingly, nasal cytology should not be regarded as an unstandardized technique. As with other morphology-based methods, residual variability related to specimen quality, sampling, and reader experience remains relevant, particularly in multicenter research. The principal next challenge is therefore not confirmation of the morphological identity of the observed cell populations, but demonstration that defined cytological patterns are reproducible across centers and add validated biological or clinical information when integrated with molecular data.

At the same time, cytological findings should not be interpreted in isolation. The presence of eosinophils, mast cells, or mixed inflammatory populations provides important information about the cellular expression of mucosal disease, but identical morphological configurations may theoretically arise from different upstream mechanisms. Accordingly, nasal cytology identifies the cellular output of inflammation, whereas endotyping aims to identify the biological programs responsible for that output.

### 7.2 Cytological phenotype is not synonymous with molecular endotype

The distinction matters. A phenotype describes observable characteristics, whereas an endotype implies a distinct biological mechanism supported by functional or molecular evidence. NARES, NARMA, and NARESMA are recognized clinical-cytological nosological entities, but their precise molecular signatures remain incompletely characterized. Their cellular composition may provide strong clues to the underlying biology, yet morphology alone cannot establish whether two apparently similar cytograms arise from identical cytokine networks, transcriptional programs, or epigenetic states.

For this reason, the term cytology-informed endotyping is preferable to “cytological endotype.” It recognizes the biological value of the cellular phenotype without assigning to morphology a mechanistic specificity that has not yet been demonstrated. Accordingly, cytology-informed endotyping is proposed here as a framework for linking observable cellular phenotypes to molecular mechanisms, rather than as an already established molecular classification.

In practical terms, nasal cytology can identify a candidate phenotype deserving deeper biological characterization. The subsequent integration of local soluble mediators, proteomic profiles, transcriptomics, epigenomics, and single-cell analyses may then determine whether that phenotype corresponds to a reproducible molecular program.

### 7.3 Proteomics and single-cell approaches: connecting morphology with molecular programs

Proteomic studies provide an intermediate layer between morphology and single-cell analysis. In seasonal AR, epithelial proteome profiling identified 133 differentially abundant proteins, with type 1 interferon signaling among the most enriched pathways and season-dependent regulation of epithelial-defense proteins (32). These data show that clinically similar allergic states can be accompanied by measurable molecular differences.

Nasal mucus proteomics independently identified disease-associated changes in proteins involved in immune responses, antiprotease balance, and epithelial permeability, further illustrating the molecular complexity underlying AR (33).

The emergence of single-cell technologies has now moved this concept to a substantially higher level of resolution. As discussed above, recent single-cell RNA and ATAC sequencing of more than one million nasal mucosal cells revealed cell-type-specific transcriptional and epigenetic abnormalities, altered epithelial differentiation, inflammatory fibroblast programs, and enhanced epithelial-stromal communication in AR (3). These approaches therefore identify the molecular programs that conventional morphology cannot resolve.

Rather than viewing cytology and single-cell omics as competing technologies, they can be considered complementary layers of analysis. Nasal cytology captures an accessible morphological output of the mucosal ecosystem, whereas single-cell and multi-omic approaches dissect the molecular programs generating that output. Linking the two levels represents a logical next step toward clinically applicable mucosal endotyping.

### 7.4 Artificial intelligence and quantitative nasal cytology

One practical limitation of conventional nasal cytology is its dependence on trained observers and manual examination of multiple microscopic fields. Digital microscopy and artificial intelligence may help reduce observer-related variability and facilitate large-scale quantitative analysis.

A recent study developed an artificial-intelligence system for quantitative assessment of nasal inflammatory cytology using whole-slide images. The system was designed to identify and quantify inflammatory cells in chronic rhinitis and represents an important step toward automated, reproducible, large-scale cytological analysis (34). The availability of automated whole-slide assessment may facilitate objective quantification, reduce observer-dependent variability, and allow much larger datasets to be linked with clinical and molecular information. At present, however, applications aimed at inferring functional interactions or molecular endotypes from cytological images remain investigational and require external validation before clinical implementation.

The potential of this transition extends beyond automated cell counting. Artificial intelligence may enable recognition of complex cellular combinations, spatial relationships, activation states, and longitudinal trajectories that are difficult to capture manually, supporting a broader move toward quantitative and precision-oriented nasal cytology (35).

The mast cell-eosinophil interaction discussed earlier provides a concrete example. Future image-analysis systems might theoretically quantify not only the abundance of each cell type but also their spatial proximity, degranulation patterns, and recurrent cellular associations. Such approaches could provide an objective means of testing whether morphological configurations such as the close mast cell-eosinophil apposition illustrated in **Figure 3** occur more frequently in particular clinical or molecular phenotypes.

### 7.5 Toward an integrated mucosal endotyping strategy

The future value of nasal cytology may therefore lie less in its use as a stand-alone diagnostic technique than in its role as the first accessible layer of a multidimensional mucosal assessment.

A possible workflow could integrate:

Clinical phenotype and allergen exposure → allergological assessment → nasal cytological phenotype → targeted local biomarkers → molecular profiling → validated mucosal endotype.

Within such a framework, cytology could help identify which patients require deeper molecular investigation, provide longitudinal monitoring at a fraction of the complexity and cost of tissue-based omics, and offer a morphological reference against which molecular signatures can be interpreted.

A proposed framework integrating clinical phenotype, allergen sensitization and exposure, nasal cytology, local biomarkers, and multi-omic information toward validated mucosal endotypes and longitudinal reassessment is summarized in **Figure 5**.

**Figure 5:**
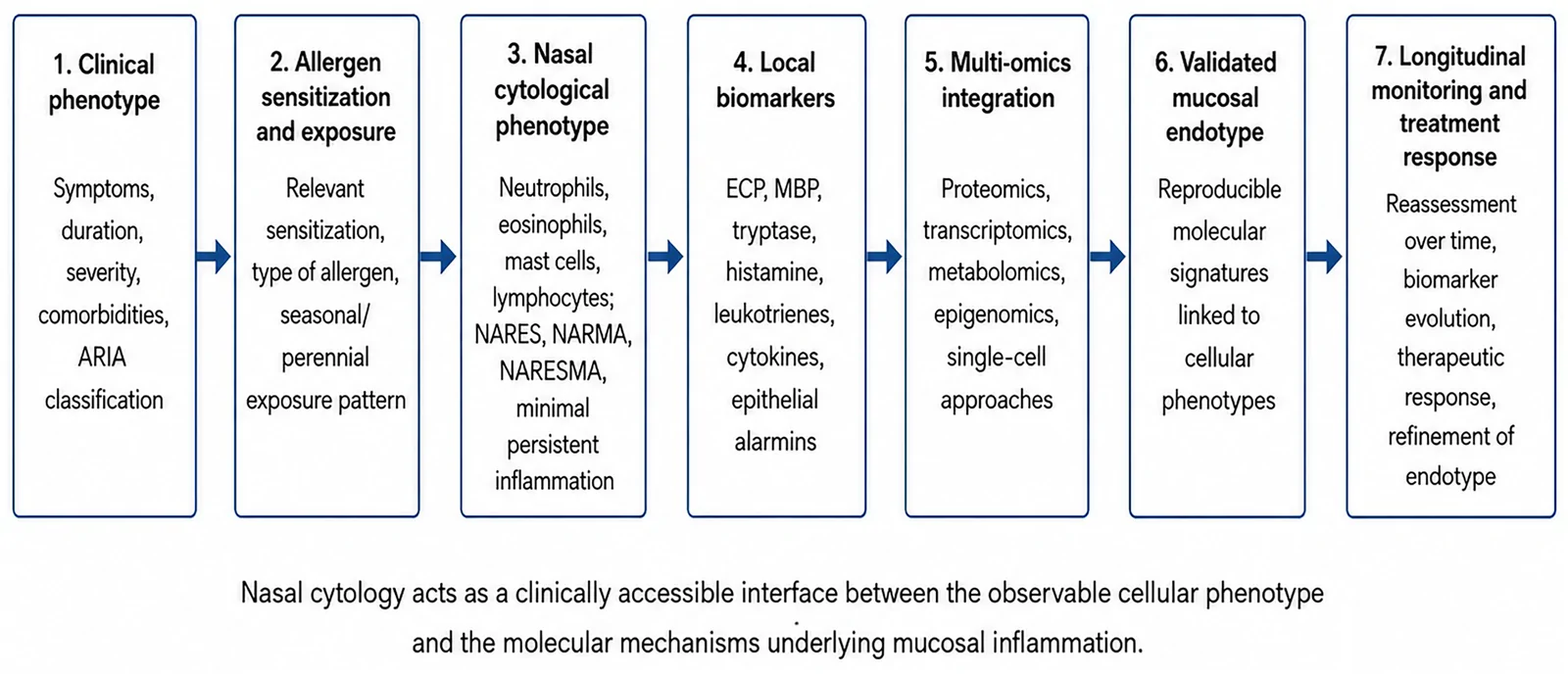
From nasal cytology to cytology-informed endotyping. Conceptual framework. The figure illustrates an integrated pathway from clinical phenotype and allergen sensitization/exposure to nasal cytological assessment, local biomarkers, multi-omics integration, and the identification of validated mucosal endotypes. Longitudinal reassessment of clinical, cytological, biomarker, and molecular features may further refine endotype assignment and support personalized treatment strategies. In this framework, nasal cytology acts as a clinically accessible interface between the observable cellular phenotype and the molecular mechanisms underlying mucosal inflammation.

This model, however, should be tested prospectively rather than assumed. Future studies must determine whether defined cytological entities correspond to stable and reproducible molecular signatures, whether those signatures persist longitudinally, and whether their identification improves prediction of disease course or therapeutic response. The objective is therefore not to replace molecular endotyping with morphology, but to use nasal cytology as a clinically accessible interface between observable mucosal inflammation and the biological mechanisms underlying it.

## 8 Clinical implications and future directions

A cytology-informed approach may refine AR assessment beyond sensitization alone. It does not replace allergological testing, nasal endoscopy, or molecular biomarkers, but can add information on the inflammatory state of the target organ, particularly when symptoms, exposure history, and standard testing are discordant.

### 8.1 Longitudinal monitoring and response to treatment

One of the main advantages of nasal cytology is its repeatability. Because sampling is minimally invasive, the same patient can be evaluated longitudinally during different phases of allergen exposure or treatment. This feature is particularly relevant in AR, where the local cellular phenotype is dynamic rather than fixed.

Preliminary clinical evidence suggests that nasal eosinophilia may change in parallel with therapeutic response. In a prospective study of 42 patients with intermittent AR undergoing subcutaneous allergen immunotherapy (SCIT), nasal eosinophil percentages, total nasal symptom scores, and nasal resistance significantly decreased after treatment, while nasal airflow improved. The reduction in nasal eosinophils correlated with symptom improvement, and higher baseline eosinophilia was associated with greater clinical benefit, particularly in grass pollen allergy (36). These findings suggest a potential role for nasal cytology both as a monitoring tool and, possibly, as a predictive biomarker, although the relatively small sample size and short observation period require cautious interpretation.

Longer-term studies of sublingual immunotherapy (SLIT) also support the concept that different biomarkers may evolve independently during treatment. In a recent study of 86 patients receiving three years of house dust mite SLIT, symptom and medication scores, endoscopic signs of mucosal inflammation, and peripheral blood eosinophil counts significantly improved, whereas total serum IgE remained unchanged (37). This dissociation illustrates an important principle: clinical improvement, local tissue changes, circulating inflammatory cells, and systemic immunoglobulin levels do not necessarily move in parallel. A multidimensional assessment may therefore provide a more accurate picture of biological response than reliance on a single biomarker.

### 8.2 Cytology and personalized treatment

Allergen immunotherapy is an established disease-modifying treatment for appropriately selected patients with IgE-mediated respiratory allergy (38). However, response to AIT is heterogeneous, and the identification of biomarkers capable of predicting or monitoring treatment efficacy remains an active area of research. Eosinophilic inflammation has been proposed as one potential biomarker domain, but currently available evidence is insufficient to support nasal eosinophil counts as a stand-alone decision tool (39).

Overlapping rhinitis provides a particularly useful example. In a patient with established AR and concomitant NARES, NARMA, or NARESMA, AIT addresses the allergen-specific component of disease but may not fully account for the associated non-allergic cellular inflammation. This does not imply failure of AIT, but rather emphasizes the need to recognize multiple simultaneously active mechanisms. In such patients, cytology may help explain why symptoms persist despite apparently appropriate allergen-directed treatment and may support a more individualized anti-inflammatory strategy.

The same principle applies to conventional pharmacotherapy. Cytological evidence of prominent eosinophilic, mast-cell, or mixed inflammation may provide additional biological context when considering intranasal corticosteroids, antihistamines, antileukotrienes, or other anti-inflammatory interventions. However, prospective interventional trials are required before treatment algorithms can be formally based on cytological patterns.

### 8.3 From association to validation

The major challenge for the next phase of research is to move from clinically meaningful associations toward formal biological validation. Several questions remain unresolved.

First, the longitudinal stability of cytological entities needs to be established across different seasons, exposure conditions, and treatment phases. A phenotype observed at a single time point may represent a transient inflammatory state rather than a stable biological trait.

Second, multicenter reproducibility must be demonstrated. The recent Delphi consensus provides an important methodological foundation, but future studies should assess inter- and intra-observer agreement, sampling reproducibility, and the performance of standardized scoring systems across independent centers.

Third, cytological phenotypes should be linked prospectively to molecular signatures. NARES, NARMA, and NARESMA provide particularly attractive models for this purpose. Transcriptomic, proteomic, metabolomic, and single-cell approaches could determine whether these recognized clinical-cytological entities correspond to reproducible biological programs or whether similar morphologies may arise through different upstream mechanisms.

Fourth, spatial relationships between cells deserve greater attention. The mast cell-eosinophil interaction discussed earlier illustrates how morphological proximity may contain biological information beyond simple cell counts. Future digital image-analysis systems could quantify cell-cell distances, degranulation patterns, and recurrent cellular associations, potentially converting qualitative observations into measurable spatial biomarkers.

Fifth, future validation should combine longitudinal cytology with matched local molecular sampling, including spatial transcriptomic or other spatial-omic approaches when feasible. Neutrophil-associated pathways also deserve attention: IL-8/CXCL8, a major neutrophil chemoattractant implicated in allergic rhinitis and chronic rhinosinusitis with nasal polyps, may provide a useful molecular axis for interpreting neutrophil-rich or mixed cytological patterns (40).

### 8.4 Toward integrated precision rhinology

The goal is not to create increasingly complex cytological classifications, but to establish which locally observable features add clinically meaningful information beyond standard allergological assessment.

In a precision-rhinology model, cytology could provide a low-cost longitudinal layer between strategically timed molecular assessments, helping identify patients in whom deeper biological characterization is most informative.

Such a strategy would allow cytology-informed endotyping to be tested rather than presumed. A cytological entity would acquire true endotypic significance only when linked to a reproducible mechanism and, ideally, to clinically relevant outcomes such as severity, natural history, treatment response, or prognosis.

## 9 Conclusions

Allergic rhinitis should no longer be interpreted solely as the local consequence of systemic allergen sensitization. The nasal mucosa represents a dynamic immunological ecosystem in which epithelial, stromal, vascular, neuronal, innate, and adaptive immune compartments interact continuously, generating local inflammatory states that may differ substantially among patients sharing the same clinical diagnosis.

Within this landscape, mast cells and eosinophils represent more than parallel effector populations. Experimental evidence supports direct physical and paracrine interactions between these cells within the allergic effector unit, and nasal cytology can provide a morphological window onto their coexistence, activation, and degranulation. The recognition of overlapping rhinitis further illustrates how an established allergic mechanism may coexist with recognized non-allergic cellular rhinitis entities, including NARES, NARMA, and NARESMA, challenging a rigid allergic versus non-allergic dichotomy.

Nasal cytology should therefore be viewed not simply as differential cell counting, but as a repeatable readout of local mucosal cellular architecture that can nominate phenotypes for deeper molecular characterization.

Cytology-informed endotyping captures this intermediate role as a proposed framework. Its validation will require multicenter studies linking morphological phenotypes with reproducible molecular signatures, longitudinal behavior, and treatment response. In this model, cytology complements rather than competes with molecular precision medicine at the target organ.
